# Immunoglobulin replacement therapies in inborn errors of immunity: a review

**DOI:** 10.3389/fped.2024.1368755

**Published:** 2024-02-15

**Authors:** Archan Sil, Suprit Basu, Vibhu Joshi, Rakesh Kumar Pilania, Sangeetha Siniah, Deepti Suri, Amit Rawat, Surjit Singh

**Affiliations:** Pediatric Allergy Immunology Unit, Department of Pediatrics, Postgraduate Institute of Medical Education and Research, Chandigarh, India

**Keywords:** immunoglobulin, inborn errors of immunity (IEI), intravenous immunoglobulin (IVIg), subcutaneous immunoglobulin (SCIg), antibody defects

## Abstract

Immunoglobulins (Ig) were used as a therapeutic modality for the first time in a patient with X-linked agammaglobulinemia in 1952 by Colonel Ogden Bruton, decades before the molecular mechanisms causing the disease were unraveled. In many autoimmune and inflammatory illnesses, human immunoglobulin has been employed as a significant immunomodulatory and immunosuppressive drug. In patients with inborn errors of immunity (IEI), immunoglobulin remains a cornerstone of management. IEIs are notable causes of recurrent infections and autoimmunity due to inheritable single-gene defects in genes encoding for different components of the immune system. As there is decreased immunoglobulin production in IEIs with antibody defects, immunoglobulin replacement is the mainstay of therapy in these disorders. Although serum immunoglobulin levels may not be low in combined immune defects, immunoglobulin replacement is still necessary in these disorders due to a deficiency of functional antibodies and qualitative defects of immunoglobulins. Commercial immunoglobulin preparations are generated from plasma donated by thousands of donors. Immunoglobulin preparations are usually available in two forms: intravenous and subcutaneous immunoglobulins. In the developed world, both intravenous immunoglobulin (IVIg) and subcutaneous immunoglobulin (SCIg) are available, and SCIg is preferred over IVIg for replacement therapy in patients with IEIs. In developing countries, IVIg remains the mainstay of replacement therapy. The rate of adverse events has significantly reduced over the last few years due to advancements in the production process. In this review article, we discuss different aspects of the use of Ig (indications, dosing, mechanism of action, route, adverse effects) in patients with IEIs.

## Background

In many autoimmune and inflammatory illnesses, human immunoglobulins have been proven to be significant immunomodulatory agents. A diverse collection of diseases known as inborn errors of immunity (IEI) can cause autoimmunity, recurring infections, and can even increase the risk of developing malignancy. IEIs are caused by heritable single gene abnormalities in the genes that code for several immune system components. To date, descriptions of more than 485 IEIs have been made (1). The first FDA-approved use of immunoglobulin replacement therapy was for IEIs (2). While immunoglobulin replacement therapy (IGRT) remains the cornerstone of management in antibody deficiency related IEI, it plays a supporting role in several other IEIs as well (3). Immunoglobulins (Ig) can be administered through intravenous and subcutaneous modes. In this review we have discussed the available preparations of immunoglobulins and their use in IEIs.

## History

It was Emil Adolf Von Behring and Shibasaburo Kitasato who first documented transfer of protection against diphtheria and tetanus in animal models in 1890 (4). Paul Ehrlich first used the word “antibody” in 1891 (5). Ig was used as a therapeutic modality for the first time in X-linked agammaglobulinemia (XLA) patient by Colonel Ogden Bruton in 1952 (6). He administered subcutaneous immunoglobulin to an 8-year-old boy who had undetectable gamma globulin fraction in his blood and suffered from repeated pneumococcal infections. Circulating gamma globulin levels improved significantly after subcutaneous immunoglobulin infusions of 3.2 g/month and complete elimination of pneumococcal infections was achieved (6). However, at that time very little was known about the molecular mechanisms of XLA.

## Principles of therapy with immunoglobulins

**Figure 1:**
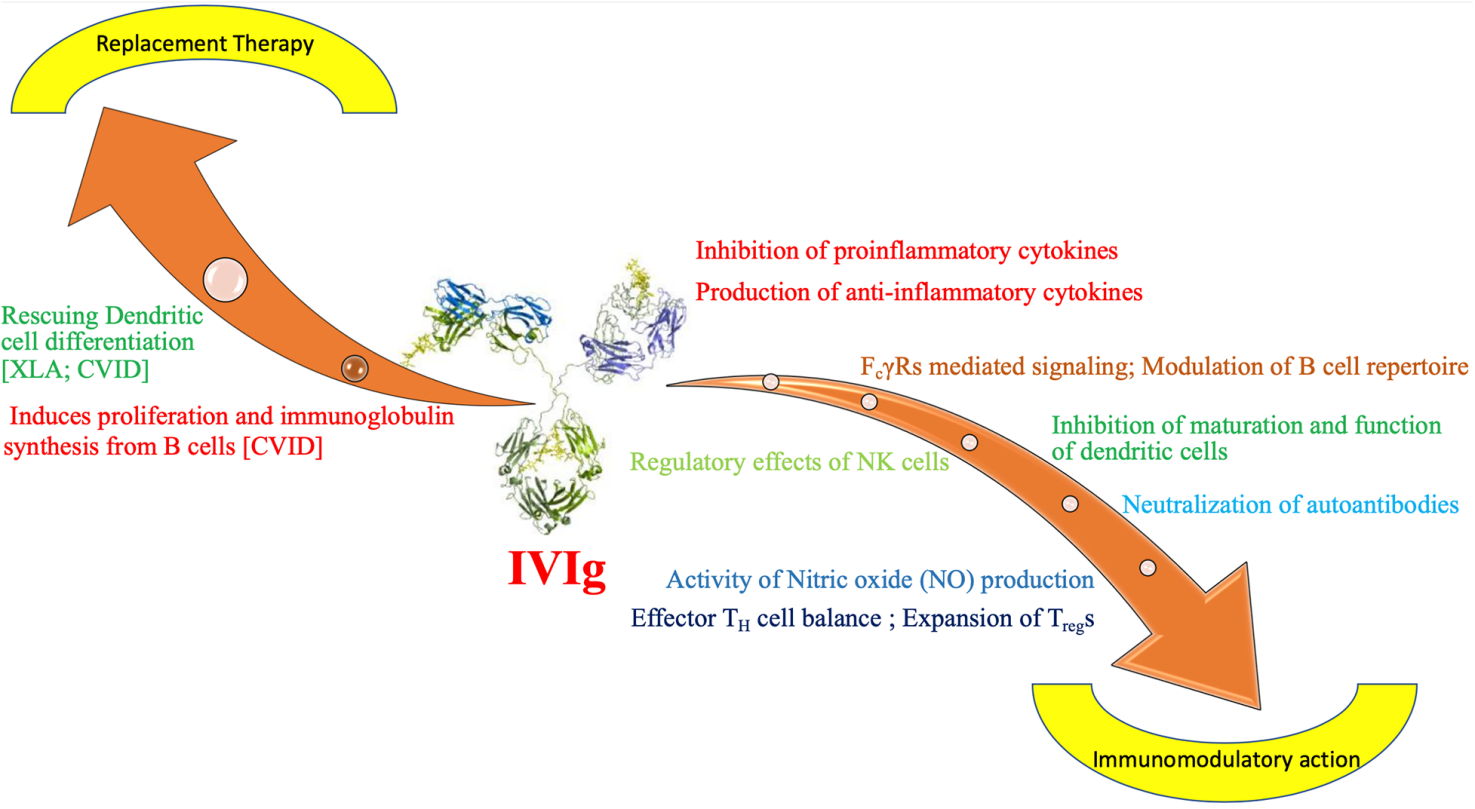
Mechanism of action of immunoglobulin as replacement and immunomodulatory therapy in inborn errors of immunity. CVID**-** Common Variable Immunodeficiency, F_c_*γ*R, F_c_ gamma receprtors; IVIg, intravenous immunoglobulin; NK, natural killer cells; T_H_, T helper cell; T_reg_, T regulatory cell; XLA, X linked agammaglobulinemia.

Ig is used in diverse clinical situations. It is often used as an immunomodulatory agent in autoimmune and inflammatory conditions. It is also used as replacement therapy (IGRT) in several IEIs. Main aim of replacement is to achieve functional levels of passive antibodies (IgG) sufficient for opsonization and neutralisation of infectious pathogens, like parasites, viruses and bacteria (5). Commonly used replacement doses are 400–600 mg/kg given every 3 weeks when used intravenously. For subcutaneous (SC) route, the dose is 100–200 mg/kg/week. For immunomodulatory effects, higher dose of Ig (1–2 g/kg) need to be given (7). Immunomodulatory action of Ig is executed by several mutually non-exclusive mechanisms. These include altering Fc receptor expression and function, controlling complement activation, affecting the cytokine network, forming antibodies against pathogens, neutralising *T*-cell superantigens, and controlling the development, activation, differentiation, and effector functions of *T* cells and B cells (8). Immunoglobulin therapy affects the growth, development, and functions of several immune system cells, including monocytes/macrophages, dendritic cells, granulocytes, NK cells, and *T* and B cells (9) (Figure 1).

### Putative mechanisms of action of Ig when used for IEIs:

1. Ig stimulates maturation and differentiation of dendritic cells (DC) in low dose. It has been observed that in patients with XLA differentiation of DC is defective (10). Ig at low dose can correct dendritic cell maturation defect and exerts a boosting effect on immune response of the host. Same enhancing effect can be observed on differentiation of DC in CVID patients (11).
2. Another important aspect of action of Ig is stimulation of B-cell proliferation and induction of immunoglobulin synthesis. It has been documented in patients with CVID (12).
3. It has important role in modulating the inflammatory manifestations of IEI by reducing secretion of inflammatory cytokines like IL-1β, IL-6 by B cells (12).
❖ Source of Immunoglobulin preparation:

Ig can be obtained from the plasma of donors undergoing plasmapheresis- this is known as “source plasma”. Ig can also be recovered from donated blood in blood banks- this is known as “recovered plasma”. **Source plasma** is directly processed from plasma donated voluntarily (usually remunerated) donors.

**Figure 2:**
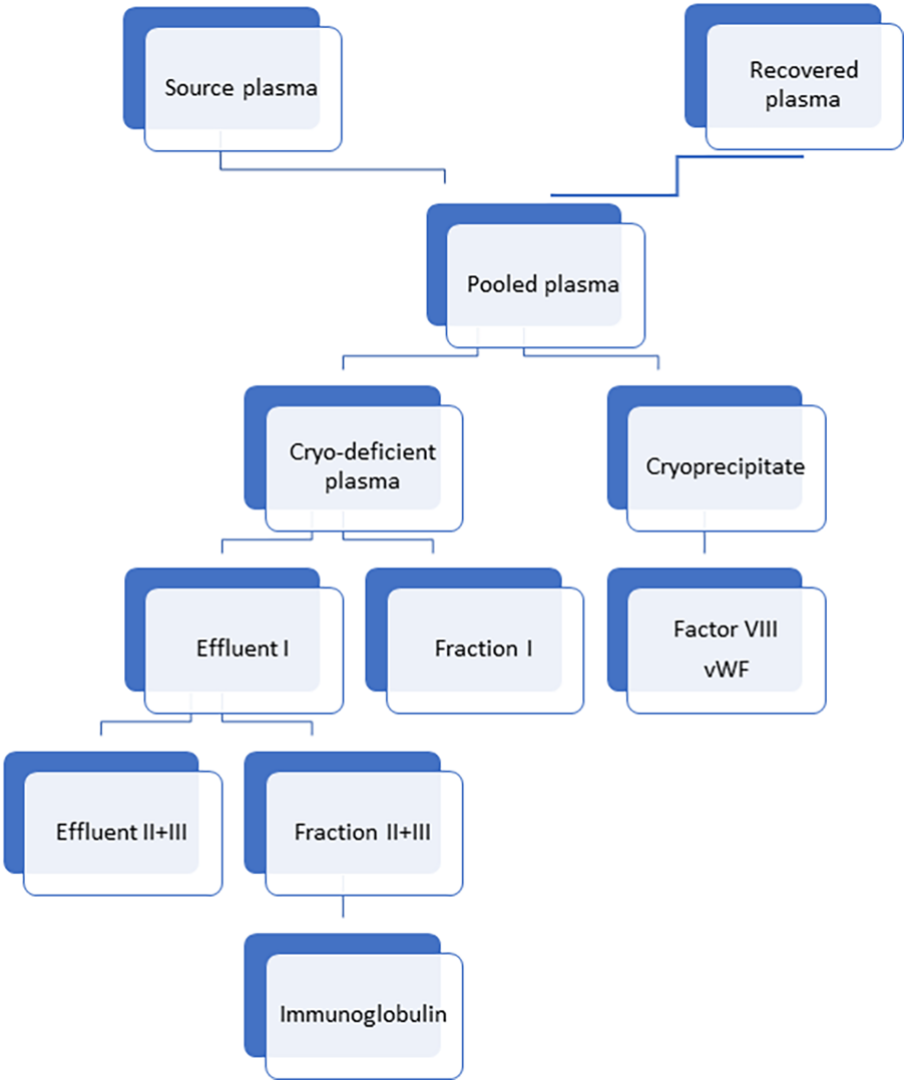
Method of preparation of immunoglobulin from different plasma sources. Cryo-deficient plasma: Cryo-deficient plasma is the plasma supernatant remaining following removal of the cryoprecipitate from frozen-thawed plasma (39).

**Recovered plasma** is a by product of whole blood separated during preparation of blood components. It is collected at blood bank from blood donated by donors meeting whole blood donor requirements (Figure 2).

❖ Donors (13):
  ▪ Selection- Selection of donor is based on the World Health Organisation (WHO) criteria:
1. Donor should be healthy (free from transfusion-transmitted infections)
2. Absence of transfusion transmitted infections [Donors should be screened for hepatitis B, C and human immunodeficiency virus (HIV) infections]
3. Appropriate interval since last donation [Donors can be divided based on the frequency of donation: (A) Frequent- Donors can donate maximum twice within a week (B) Infrequent- Donors can donate once every month or less]
4. Physical assessment of the donor: Parameters like body weight, pulse, blood pressure, temperature should be checked and haemoglobin level should also be measured.
  ▪ Testing details: Testing should be carried out for syphilis, malaria, cytomegalovirus, HIV, hepatitis B surface antigen (HBsAg), and hepatitis C virus (HCV) serology. It is recommended to utilise molecular (polymerase chain reaction) and serological approaches. Additionally advised testing for retrieved plasma includes human T-lymphotropic virus (HTLV) I and II, West Nile virus, and Chagas disease.
❖ Storage of plasma

Source plasma should be stored at a temperature lesser than −20°C. Regulation for minimum freezing temperature has been set by Food and Drug Administration (FDA). Protein activity can be preserved by rapid freezing following plasmapheresis.

In source plasma, donors are screened for infections and if normal, only then their plasma is considered. Units of plasma are then tested for known blood-borne diseases and if found positive, is discarded. As per FDA 60 days “quarantine” or “inventory hold” procedure is done where donor plasma is stored separately until the donor returns to provide another unit of plasma. Only if the second and subsequent donations test negative then the previous units are released. Tracking of all products can be done with the help of computerized databases. Ig preparations are derived from pooled plasma of 1,000–100,000 healthy donors. Use of such a high number of plasma donors ensure that there is broad spectrum of protective antibodies. IgG fraction is the main component with very low IgA and IgM. The concentration of IgA fraction varies from 0.6–40 mg per 100 ml depending on the preparation (14). Half-life of IgG is 3–4 weeks. Both IVIg and SCIg preparation have IgG1, IgG2, IgG3 and IgG4 levels comparable to human plasma. IgG molecules tend to aggregate in concentrated solution bringing the Fc fragment into proximity. These Fc portions cause activation of complement and cross-linking of Fc*γ*-receptors. As a result there is production of inflammatory mediators which lead to adverse events during IgG infusions (15). To prevent IgG aggregation, sugar stabilizers are used like sorbitol, glucose, or maltose. Recently, amino acids like proline or glycine are preferred as stabilizers because osmotic tubular injury has been reported with Ig stabilising sugar components (14, 16). Purification process can adversely affect the quality and biological activity of immunoglobulins in terms of efficacy and safety. There is variation in content of salt and IgG concentration, and not all products are licensed for use by different routes. So route of Ig administration should be individualized. Unpredictable idiosyncratic reactions can occur due to use of large donor pools, leading to difference in specific antibody and plasma protein concentration.

Immunoglobulins play a pivotal role in humoral adaptive immunity; IVIg reflects a collective exposure of the donor population to their environment and can be expected to contain an antibody repertoire of multiple specificities against a broad spectrum of infectious agents (bacterial, viral, and others), self-antigens and anti-idiotype antibodies.

### Types of immunoglobulin preparation

Immunoglobulin preparations are usually available in 3 forms –

1. Intravenous immunoglobulin (IVIg)
2. Subcutaneous immunoglobulins (SCIg).
3. SCIg with hyaluronidase facilitated

There are reports of use of intramuscular IgG (IGIM) in the 1950s, 60s and 70s. However, it was associated with adverse events like chronic pain at the injection site, hypotension, unconsciousness, tightness in the chest, dyspnea, and episodes of facial swelling. Children with low muscle mass used to have difficulty receiving IGIM, necessitating more frequent injections to attain adequate IgG levels. Immunoglobulin preparations are to be used at frequent intervals unlike specific immunoglobulin like tetanus anti-toxin which is mostly used on single occasion. Therefore, its use became obsolete due to pain at the injection site, frequent dosing, and poor compliance (17).

While IVIg preparations are available in 5% and 10% concentrations, SCIg as 10%, 16.5%, and 20% solutions (Table 1). In developed world both IVIg and SCIg are available. Most European countries and the USA SCIg is preferred over IVIg for IGRT in patients with IEIs. In developing world IVIg still remains mainstay of IGRT. Different commercially available FDA approved preparations of immunoglobulin have been mentioned in Table 1.

**Table 1:** Commercially available FDA approved preparations of immunoglobulin.

Name of brands	Available concentrations	Reconstitution	Routes of administration	IgA content	Sugar content
Gammagard	5% and 10%	Required- available in powder form	Intravenous	≤2.2 µg/ml	Glucose added
Gammagard liquid	10%	Not required- Available in liquid form	Intravenous/ subcutaneous	37 µg/ml	Nil
Carimune	3%, 6%, 12%	Required- available in powder form	Intravenous	720 µg/ml	Sucrose added
Bivigam	10%	Not required- Available in liquid form	Intravenous	≤200 µg/ml	Nil
Flebogamma	5% and 10%	Not required- Available in liquid form	Intravenous	<3 µg/ml	Nil
Octagam	5% and 10%	Not required- Available in liquid form	Intravenous	<100 µg/ml	Maltose added
Gammaplex	5% and 10%	Not required- Available in liquid form	Intravenous	<4 µg/ml for 5% and <20 µg/ml for 10%	D-Sorbitol present in 5% solution
Privigen	10%	Not required- Available in liquid form	Intravenous	≤25 µg/ml	Nil; Stabilized with Proline
Gammaked	10%	Not required- Available in liquid form	Intravenous/Subcutaneous	46 µg/ml	Nil
Gamunex-C	10%	Not required- Available in liquid form	Intravenous/Subcutaneous	46 µg/ml	Nil

## Subcutaneous immunoglobulin vs. intravenous immunoglobulin

First SCIg in treatment of IEI was used in 1952 (6). Bruton treated his patient with SCIG replacement at “20 cc. containing 3.2 g of gamma globulin” and continued with monthly doses leading to resolution of recurrent infection in the patient (18).

But after that intramuscular immunoglobulin initially, and IVIg later was the standard of care until 1995 and 2006 when SCIg was approved in Europe and United States respectively (18). An international crossover trial that was conducted in 2000 showed that SCIG was not inferior to IVIG, and further research showed that SCIG is as effective in IEIs (18). Enzyme facilitated subcutaneous immunoglobulin (fSCIG) was approved in Europe in 2015 and in the US in 2016 (18).

## Advantages of SCIg over other forms

Due to several advantages SCIg has become a standard of care in IEIs. Ease of administration, home-based therapy, no loss of working days, no need of vascular access, low adverse events and maintenance of steady state of IgG level are the advantages of SCIg over IVIg. It is also helpful in patients having unacceptable side-effects with IVIg or whom venous access is difficult. Small and more frequent doses of SCIg help to achieve steady-state level of IgG which provides better protection against infections (19) (Table 2).

**Table 2:** Comparison of intravenous and subcutaneous immunoglobulin.

Characteristics	Intravenous	Subcutaneous
Frequency of infusion	Every 3–4 weeks	Usually given once in 7–10 days
Duration of infusion	Longer (at least over 6–8 h)	Shorter (2–4 h)
Administration of large volume	Is not difficult	Only a limited volume can be given in one sitting
Available concentrations	5%, 10%	10%, 16.5%, and 20%
Pharmacokinetics	Rapid increase initially followed by wearing off effect by 3^rd^–4^th^ week	Steady serum IgG level. No wearing of effects
Dosing schedule	400–600 mg/kg every 3–4 weeks	100–200 mg/kg/week
Advantages	a. Proven efficacy b. Better monitoring	a. Home administration b. Venous access not required c. Systemic side effects are minimal d. Less needle discomfort and pain e. Less requirement of premedications
Disadvantages	a. Venous access is essential b. It requires hospital admission c. Chances of acute febrile reactions, headache and aseptic meningitis are higher d. Trough levels	a. Local site reactions like erythema, induration or swelling b. More frequent infusions are required

## Situation where IVIg is preferred over SCIg

If patient or caretaker is physically incapable of administering the infusion or the patient is having severe thrombocytopenia, bruising, bleeding at the sites of infusion or having severe skin diseases then IVIg is preferred to SCIg. However, it is important to note that in a given patient with IEI, choice of Ig should be individualized, and decided after detailed discussion with parents and caregivers (18).

## Dosage of IVIg

Dose should be individualised for IGRT and should be titrated to achieve best clinical effect. Dose is best determined by the level required to remain “infection free” (20). Average half-life of total IgG is 25.8 days and the half-life for IgG1, IgG2 and IgG3 was found to be 29.7, 26.9 and 15.7 days respectively (21). The values are consistent with those reported for endogenous IgG, but there are major differences in IgG metabolism. Higher IVIg dosages (400–600 mg/kg vs. 100–200 mg/kg q 3–4 weeks) have been shown to be superior in studies on patients with primary hypogammaglobulinemia for lowering infection rates, reducing hospitalizations, reducing antibiotic use, and improving pulmonary outcomes. The recommended dosage of IVIg replacement is therefore 400–600 mg/kg of body weight every three weeks. IVIg dose needs to be titrated to maintain the trough level >500 mg/dl just before the next infusion (22).

## Dosage of SCIg

Seven to ten days following the last dose of IVIg, the first dose of SCIg is administered. The dose is at least the same dose of maintenance IVIg therapy and infusion rate is 15 ml/hour/site. Rate of subcutaneous infusion can be increased to 25 ml/hour/site (maximum −50 ml/hour/site) and a maximum of 4–8 sites can be infused at one time. In SCIG, Ig levels remain stable and trough level is also higher than IVIg. There are 2 methods of SCIg application −1] conventional (using infusion pump—rapid-push SCIG) 2], facilitated (fSCIG)(initial administration of human recombinant hyaluronidase in the same needle as IgG) (23). Home SCIG is affordable, secure, therapeutically beneficial, and frequently favoured by patients and medical professionals. Since just a little amount of IgG can be pumped into the subcutaneous tissue, conventional SCIG needs weekly infusion. However, like IVIg, fSCIG can be taken every 4 weeks and delivers a higher dose of IgG (24). It reduces burden of SCIg treatment, improve patients’ quality of life and compliance to therapy (25) (Table 2).

## Monitoring during transfusion

IVIg should be started at minimal rate and under close observation during first transfusion. Monitoring of vital signs is important. Infusion rate can be doubled every 15–30 min if the patient is stable to a peak of @ 0.08 ml/kg/minute (4 mg/kg/min of 5% or 8 mg/kg/min of 10% solution) (Table 3) (26).

**Table 3:** Checklist to follow during administration of IVIg.

Checklist	1. Consent and counseling regarding adverse events during and after transfusion. 2. Calculate the dose of IVIg to be given; check batch number and expiry date. 3. Intravenous line to be started with running normal saline drip. 4. Prefilled syringes with adrenaline, hydrocortisone with proper labels of dose should be kept ready at bed side. 5. Starting dose is 0.1–0.2 ml/kg/hr for 15 min, then increase every 15 min. Rate of infusion should not exceed 2 ml/kg/hr in the first hour of infusion. 6. Monitor vital parameters (heart rate, respiratory rate, blood pressure and SpO_2)_ every 15 min for first hour and then half hourly for the rest of the period. 7. If the child is clinically stable, final rate of infusion can be increased to 20 ml (smaller child) to 60 ml (older child) per hour. 8. Duration of infusion: in day care regular infusion of 400–800 mg/kg over 3–4 h. The rate should be slower if the child develops any mild reaction to it (like chills, fever). In high dose infusions (2 mg/kg): over 8–12 h; in some situations like small children, cardiovascular instability and mild infusion related reactions as stated above duration may exceed to 18- 20 h. 9. If anaphylaxis develops: stop the drip immediately, give oxygen and inject adrenaline (0.1 mg/kg of 1:1,000) and hydrocortisone (10 mg/kg) immediately. Do not restart again. For minor reactions like fever, chills, headache: infusion should be withheld and then restarted at a slower rate.

## Adverse effects of immunoglobulin

The use of IMIg is restricted by the discomfort and hazards involved with intramuscular injections. Infusion-related adverse effects are more common in treatment naïve cases and in those who are on irregular therapy or are chronically infected. This is caused by the rapid release of lipopolysaccharides or other components of pathogens that are already present in the recipient, as well as the creation of antigen-antibody complexes while immunoglobulin is administered (15, 26).

## Common adverse reactions

Common adverse events are chills and rigors, arthralgias, myalgias, headache, anaphylaxis and anaphylactoid reactions. These occur in less than 10% of the cases.

### Management of headache

Headaches can occur up to 48–72 h of infusion. Headache may be similar to that of migraine in character or in some rare case associated with meningismus, and aseptic meningitis. Adequate fluid intake should be ensured and paracetamol can be used (15).

### Methods to reduce infusion related adverse events:

1. This reaction risk can be reduced by transfusing the patient in an afebrile and infection-free state.
2. Incidence of reactions increase if patient receive different brands of IVIg (27). Hence, it is preferrable to continue with the same brand.
3. Rate-related side-effects can be minimised by temporary stoppage of infusion or slowing the rate of infusion [@ 0.01 ml/kg/minute (=0.5 mg/kg/minute of 5% solution; 1 mg/kg/minute of 10% solution)].
4. If these symptoms persist antipyretics, antihistaminics may help.

## Anaphylactoid reactions

Anaphylactoid reactions are immediate systemic reactions that have similarities with anaphylaxis but are not mediated by IgE. These can occur even without prior exposure. Anaphylactic reactions are IgE mediated and occur only with prior exposure. It is seen in some patients, who complain of chest tightness, anxiety and a feeling of impending doom and is associated with flushing and tachycardia.

## Precautions that can be taken to reduce risk of anaphylaxis

Any medical professional or setting that delivers IVIg needs to be adequately prepared to handle anaphylaxis.

## Rare complications

Transfusion-related acute lung injury, thromboses and acute kidney injury are some other rare complications (26).

**Thrombotic events**, major side-effects of immunoglobulin treatment have an estimated incidence of 1%–16.9% (28, 29). In a survey of thrombotic adverse events made between 2008 and 2010, Daniel et al. discovered that 1.2 percent (122/11,785) of the patients experienced immunoglobulin-induced thrombotic events. The majority (80%) of thrombotic events occurred within 24 h of the end of immunoglobulin delivery and were stroke and myocardial infarction (28). An increase in plasma viscosity, the activation of procoagulant factors, vasospasm, autoimmune vasculitis, and an elevated platelet count are some of the mechanisms that could cause thrombotic events. Contact activation of clotting factors [coagulation factor F(XI)] with Ig during product manufacturing processes may lead to an elevated thrombotic potential.

**Renal dysfunctions** are rare with the commonest being proximal tubular damage. Majority of renal side effects were due to osmotic effects secondary to sugar (sucrose) used in older preparations of therapeutic immunoglobulin.

It is often seen that a patient may have adverse effects with one particular brand and tolerate another brand relatively well. In these cases switching of brand is indicated but infusions should be initially started at a slow infusion rate.

## Side effects encountered during SCIg infusion

SCIg infusion rarely causes side-effects or significant vital sign changes. Subcutaneous injections may carry a risk of cellulitis or local site infections at infusion sites. Most patients will have swelling and redness (30).

## Management of the side effects

Before starting infusion, blood return is to be checked to prevent inadvertent intravascular administration. Injected fluid may cause fluctuance and differentiating from abscess can be difficult. The infused medicine is dispersed via increased local circulation brought on by a warm compress or light massage. Within hours after the infusion's completion, the majority of local responses at the infusion site disappear. A healthcare professional's opinion is required if the infusion site exhibits redness, warmth, or swelling that gets worse over time, suggesting an infection (27). These patients ought to be able to get in touch with an on-call doctor or nurse. The patient can use a pen to indicate the size of their local reaction and objectively monitor any prospective growth (15).

## Advantages of subcutaneous form in patients with cardiovascular, thrombotic and renal complications

IgG is administered slowly with most subcutaneous regimens, and adsorption from the subcutaneous site is slower than with IV infusions. The total monthly dose is divided into four distinct doses. SCIg is therefore preferable in patients with renal, thrombotic, or cardiovascular problems (30).

### Indications of immunoglobulin in IEI

The indications of using immunoglobulin in IEI has widened (31). Antibody deficiencies are the commonest type of IEI described worldwide. According to literature, they account for almost half of the cases of IEI reported. Some of the studies have reported the proportion of antibody deficiencies as high as 78% (32). As there is decreased immunoglobulin production in IEIs with antibody defects, the primary modality of treatment in these disorders is IGRT. Regular IGRT is the mainstay of therapy in humoral immunodeficiencies like XLA, CVID, hyper IgM syndrome and IgG subclass deficiencies. IGRT is also essential in severe combined immunodeficiency (SCID). Although serum immunoglobulin levels may not be low in combined immune defects like autosomal dominant STAT-3 loss of function, autosomal recessive DOCK-8 deficiency, or Wiskott Aldrich Syndrome (WAS), immunoglobulin replacement is still necessary in these disorders due to deficiency of functional antibodies and qualitative defects of immunoglobulins. IGRT is usually required in patients with combined immune defect while awaiting hematopoietic stem cell transplantation (HSCT). However, in patients with milder impairment of *T* cell function, IGRT may remain an important therapeutic armamentarium. IEIs can be divided into different phenotypes for that IGRT is indicated (2). These are:

(1) **Agammaglobulinemia**- This is the definitive indication of Ig replacement. The prototype of this category is XLA. Regular IVIg supplementation showed significant reduction in both acute and chronic infections in patients with B-cell deficiency (33). It has been observed that severe bacterial infections and enteroviral meningoencephalitis can be avoided if IgG trough levels are kept above 800 mg/dl (33). It has been seen that children with primary antibody deficiency like XLA (on regular IGRT) have good prognosis in developing countries in spite of economic and other challenges. Mean trough IgG levels are reported to be much lower in developing nations than Western population (20).
(2) **Hypogammaglobulinemia with impaired antibody function**- This group of disorders are characterised by either decreased production and/or defective response with IgG antibody on antigen challenge. The prototype of this category is CVID. IVIg transfusion in these patients showed decreased prevalence of infections in comparison to the infection rates prior to initiation of IVIg (34) and in long run chronic lung changes and autoimmune complications are also found to be less in these patients (35). B cell proliferation and antibody production are induced by IVIg in low doses. IVIg replacement therapy in CVID induces B cell activation and proliferation in T cell independent manner. Anti-inflammatory action of IVIg is mediated by suppression of production of inflammatory cytokines from B cells. Therefore, IVIg, as a treatment modality, not only aids in replacement of antibody, but also plays an important role in immunomodulation (12).
(3) **Hypogammaglobulinemia with normal antibody function**- Although IgG levels normalize with increasing age in patients with transient hypogammaglobulinemia of infancy, initially antibody function may be partially impaired, requiring treatment with immunoglobulin replacement (36).
(4) **Combined Immunodeficiency**- Studies have demonstrated that IGRT decrease rates of infections and antibiotic use, and improve quality of life even in patients with combined immunodeficiency (37).
(5) **Normal immunoglobulins with impaired antibody function**- Immunoglobulin therapy should be initiated in patients who have normal total IgG levels, but poor response to polysaccharide antigens following vaccination due to defective specific antibody production (38).
(6) **Autoimmunity in IEI**- In primary immunodeficiencies with autoimmunity, IVIg therapy may be beneficial (1). For example, autoimmune cytopenias in Wiskott Aldrich Syndrome shows good response to immunoglobulin treatment. The mechanism and dose of IVIg in autoimmune diseases are not based on good quality evidences. Some anecdotal reports are the principal sources.

Continuously changing mode of immunoglobulin therapy is a life-saving option for patients with IEI, especially those with antibody deficiency and immune dysregulation. Considering the ever-increasing demand of Ig preparations for IEIs, more clinical trials and research projects at the basic level are required for better understanding of mechanisms of action and use of Ig. SCIg is a relatively newer modality of IGRT in developing countries, which is now being increasingly used in clinical practice (Table 4).

**Table 4:** Indications of immunoglobulin in inborn errors of immunity.

A. Quantitative Ig deficiency	1. Agammaglobulinemia [eg., X-linked Agammaglobulinamia (XLA)] 2. Hypogammaglobulinemia with poor antibody function [eg. Common Variable Immunodeficiency (CVID)] 3. Hypogammaglobulinemia with normal antibody function (eg. Transient hypogammaglobulinemia of infancy) 4. Combined Immunodeficiency [eg., Severe Combined Immunodeficiency (SCID)]
B. Qualitative Ig deficiency	1. Poor antibody function (eg. Hyper Ig E Syndrome)
C. IEI with autoimmunity	1. Wiskott Aldrich Syndrome 2. Common Variable Immunodeficiency (CVID)

## References

[1] Tangye SG Al-Herz W Bousfiha A Cunningham-Rundles C Franco JL Holland SM Human inborn errors of immunity: 2022 update on the classification from the international union of immunological societies expert committee. J Clin Immunol. (2022) 42(7):1473–1507. 10.1007/s10875-022-01289-3 35748970 PMC9244088

[2] Perez EE Orange JS Bonilla F Chinen J Chinn IK Dorsey M Update on the use of immunoglobulin in human disease: a review of evidence. J Allergy Clin Immunol. (2017) 139(3):S1–46. 10.1016/j.jaci.2016.09.023 28041678

[3] Matucci A Maggi E Vultaggio A. Mechanisms of action of Ig preparations: immunomodulatory and anti-inflammatory effects. Front Immunol. (2015) 5:690. 10.3389/fimmu.2014.00690 25628625 PMC4290674

[4] von Behring E Kitasato S. The mechanism of diphtheria immunity and tetanus immunity in animals. 1890. Mol Immunol. (1991) 28(12):1317. 1319–20. 10.1016/0161-5890(91)90032-F 1749380

[5] Lindenmann J. Senior overviews. Scand J Immunol. (1984) 19(4):281–5. 10.1111/j.1365-3083.1984.tb00931.x 6374880

[6] Bruton OC. Agammaglobulinemia. Pediatrics. (1952) 9(6):722–8. 10.1542/peds.9.6.722 14929630

[7] Durandy A Kaveri SV Kuijpers TW Basta M Miescher S Ravetch JV Intravenous immunoglobulins—understanding properties and mechanisms. Clin Exp Immunol. (2009) 158(Supplement_1):2–13. 10.1111/j.1365-2249.2009.04022.x 19883419 PMC2801035

[8] Kazatchkine MD Kaveri SV. Immunomodulation of autoimmune and inflammatory diseases with intravenous immune globulin. N Engl J Med. (2001) 345(10):747–55. 10.1056/NEJMra993360 11547745

[9] Kaveri SV Maddur MS Hegde P Lacroix-Desmazes S Bayry J. Intravenous immunoglobulins in immunodeficiencies: more than mere replacement therapy. Clin Exp Immunol. (2011) 164(Supplement_2):2–5. 10.1111/j.1365-2249.2011.04387.x 21466545 PMC3087903

[10] Bayry J Lacroix-Desmazes S Donkova-Petrini V Carbonneil C Misra N Lepelletier Y Natural antibodies sustain differentiation and maturation of human dendritic cells. Proc Natl Acad Sci USA. (2004) 101(39):14210–5. 10.1073/pnas.0402183101 15381781 PMC521138

[11] Bayry J Lacroix-Desmazes S Hermine O Oksenhendler E Kazatchkine MD Kaveri SV. Amelioration of differentiation of dendritic cells from CVID patients by intravenous immunoglobulin. Am J Med. (2005) 118(12):1439–40. 10.1016/j.amjmed.2005.06.028 16378810

[12] Bayry J Fournier EM Maddur MS Vani J Wootla B Sibéril S Intravenous immunoglobulin induces proliferation and immunoglobulin synthesis from B cells of patients with common variable immunodeficiency: a mechanism underlying the beneficial effect of IVIg in primary immunodeficiencies. J Autoimmun. (2011) 36(1):9–15. 10.1016/j.jaut.2010.09.006 20970960

[13] Weinstein M. Regulation of plasma for fractionation in the United States. Ann Blood. (2018) 3:3–3. 10.21037/aob.2017.12.02

[14] Guilpain P Kaveri SV Mouthon L. Autoantibodies in therapeutic preparations of human intravenous immunoglobulin (IVIG). In: Shoenfeld Y Gershwin ME Meroni PL, editors. Autoantibodies. 2nd Edn. Elsevier (2007). p. 293–8. Available online at: https://linkinghub.elsevier.com/retrieve/pii/B9780444527639500433

[15] Berger M. Principles of and advances in immunoglobulin replacement therapy for primary immunodeficiency. Immunol Allergy Clin North Am. (2008) 28(2):413–37. 10.1016/j.iac.2008.01.008 18424340 PMC7127239

[16] Stacy Ness PD. Intravenous and subcutaneous immunoglobulin treatment options. In: Examining the Application of Immunoglobulin in Multiple Disease States: A Review of Evidence. Supplements and Featured Publications (2019). Vol. 25, No. 6, p. S0. Available online at: https://www.ajmc.com/view/intravenous-and-subcutaneous-immunoglobulin-treatment-options (cited April 5, 2022).

[17] Wasserman RL. Progress in gammaglobulin therapy for immunodeficiency: from subcutaneous to intravenous infusions and back again. J Clin Immunol. (2012) 32(6):1153–64. 10.1007/s10875-012-9740-x 22828788

[18] Kobayashi RH Litzman J Rizvi S Kreuwel H Hoeller S Gupta S. Overview of subcutaneous immunoglobulin 16.5% in primary and secondary immunodeficiency diseases. Immunotherapy. (2022) 14(4):259–70. 10.2217/imt-2021-0313 34986666

[19] Duff C Ballow M. Nuts and bolts of subcutaneous therapy. Immunol Allergy Clin North Am. (2020) 40(3):527–37. 10.1016/j.iac.2020.04.002 32654697

[20] Suri D Bhattad S Sharma A Gupta A Rawat A Sehgal S Serial serum immunoglobulin G (IgG) trough levels in patients with X-linked agammaglobulinemia on replacement therapy with intravenous immunoglobulin: its correlation with infections in Indian children. J Clin Immunol. (2017) 37(3):311–8. 10.1007/s10875-017-0379-5 28321612

[21] Mankarious S Lee M Fischer S Pyun KH Ochs HD Oxelius VA The half-lives of IgG subclasses and specific antibodies in patients with primary immunodeficiency who are receiving intravenously administered immunoglobulin. J Lab Clin Med. (1988) 112(5):634–40. . 3183495

[22] Prasad AN Chaudhary S. Intravenous immunoglobulin in pediatrics: a review. Med J Armed Forces India. (2014) 70(3):277–80. 10.1016/j.mjafi.2013.05.011 25378784 PMC4213893

[23] Wiesik-Szewczyk E Sołdacki D Paczek L Jahnz-Różyk K. Facilitated subcutaneous immunoglobulin replacement therapy in clinical practice: a two center, long-term retrospective observation in adults with primary immunodeficiencies. Front Immunol. (2020) 11:981. 10.3389/fimmu.2020.00981 32670265 PMC7326142

[24] Pulvirenti F Cinetto F Pecoraro A Carrabba M Crescenzi L Neri R Health-related quality of life in patients with CVID under different schedules of immunoglobulin administration: prospective multicenter study. J Clin Immunol. (2019) 39(2):159–70. 10.1007/s10875-019-0592-5 30644015 PMC6445807

[25] Carne E Ponsford M El-Shanawany T Williams P Pickersgill T Jolles S. Five years of self-administered hyaluronidase facilitated subcutaneous immunoglobulin (fSCIg) home therapy in a patient with primary immunodeficiency. J Clin Pathol. (2016) 69(1):87–8. 10.1136/jclinpath-2015-202901 26188056

[26] Orbach H Katz U Sherer Y Shoenfeld Y. Intravenous immunoglobulin: adverse effects and safe administration. Clin Rev Allergy Immunol. (2005) 29(3):173–84. 10.1385/CRIAI:29:3:173 16391392

[27] Berger M Pinciaro PJ. Flebogamma 5% investigators. Safety, efficacy, and pharmacokinetics of flebogamma 5% [immune globulin intravenous (human)] for replacement therapy in primary immunodeficiency diseases. J Clin Immunol. (2004) 24(4):389–96. 10.1023/B:JOCI.0000029108.18995.61 15163895

[28] Daniel GW Menis M Sridhar G Scott D Wallace AE Ovanesov MV Immune globulins and thrombotic adverse events as recorded in a large administrative database in 2008 through 2010. Transfusion. (2012) 52(10):2113–21. 10.1111/j.1537-2995.2012.03589.x 22448967

[29] Guo Y Tian X Wang X Xiao Z. Adverse effects of immunoglobulin therapy. Front Immunol. (2018) 9:1299. 10.3389/fimmu.2018.01299 29951056 PMC6008653

[30] Kobrynski L. Subcutaneous immunoglobulin therapy: a new option for patients with primary immunodeficiency diseases. Biologics. (2012) 6:277–87. 10.2147/BTT.S25188 22956859 PMC3430092

[31] Krivan G Jolles S Granados EL Paolantonacci P Ouaja R Cissé OA New insights in the use of immunoglobulins for the management of immune deficiency (PID) patients. Am J Clin Exp Immunol. (2017) 6(5):76–83. . 29181272 PMC5698561

[32] Patel NC. Individualized immunoglobulin treatment in pediatric patients with primary humoral immunodeficiency disease. Pediatr Allergy Immunol. (2018) 29(6):583–8. 10.1111/pai.12923 29744952

[33] Quartier P Debré M De Blic J de Sauverzac R Sayegh N Jabado N Early and prolonged intravenous immunoglobulin replacement therapy in childhood agammaglobulinemia: a retrospective survey of 31 patients. J Pediatr. (1999) 134(5):589–96. 10.1016/S0022-3476(99)70246-5 10228295

[34] Busse PJ Razvi S Cunningham-Rundles C. Efficacy of intravenous immunoglobulin in the prevention of pneumonia in patients with common variable immunodeficiency. J Allergy Clin Immunol. (2002) 109(6):1001–4. 10.1067/mai.2002.124999 12063531

[35] de Gracia J Vendrell M Álvarez A Pallisa E Rodrigo MJ de la Rosa D Immunoglobulin therapy to control lung damage in patients with common variable immunodeficiency. Int Immunopharmacol. (2004) 4(6):745–53. 10.1016/j.intimp.2004.02.011 15135316

[36] Dorsey MJ Orange JS. Impaired specific antibody response and increased B-cell population in transient hypogammaglobulinemia of infancy. Ann Allergy Asthma Immunol. (2006) 97(5):590–5. 10.1016/S1081-1206(10)61085-X 17165264

[37] Abrahamian F Agrawal S Gupta S. Immunological and clinical profile of adult patients with selective immunoglobulin subclass deficiency: response to intravenous immunoglobulin therapy. Clin Exp Immunol. (2010) 159(3):344–50. 10.1111/j.1365-2249.2009.04062.x 20015274 PMC2819500

[38] Orange JS Ballow M Stiehm ER Ballas ZK Chinen J De La Morena M Use and interpretation of diagnostic vaccination in primary immunodeficiency: a working group report of the basic and clinical immunology interest section of the American academy of allergy, asthma & immunology. J Allergy Clin Immunol. (2012) 130(3):S1–24. 10.1016/j.jaci.2012.07.002 22935624

[39] Sparrow RL Simpson RJ Greening DW. A protocol for the preparation of cryoprecipitate and cryo-depleted plasma for proteomic studies. Methods Mol Biol. (2017) 1619:23–30. 10.1007/978-1-4939-7057-5_2 28674874

